# 3D-Bioreactor culture of human hepatoma cell line HepG2 as a promising tool for *in vitro* substance testing

**DOI:** 10.1186/1753-6561-5-S8-P61

**Published:** 2011-11-22

**Authors:** Christiane Goepfert, Wibke Scheurer, Susanne Rohn, Britta Rathjen, Stefanie Meyer, Anja Dittmann, Katharina Wiegandt, Rolf Janßen, Ralf Pörtner

**Affiliations:** 1 Institute of Bioprocess and Biosystems Engineering, Hamburg University of Technology Hamburg, D-21073, Germany; 2 Institute of Advanced Ceramics, Hamburg University of Technology, Hamburg, D-21073, Germany

## Introduction

Future developments in pharmaceutical research and regulatory requirements such as the European REACH program require high numbers of animal experiments. As a result of ethical concerns, cell culture tests with human cell lines or primary cells are considered as an alternative. However, current testing protocols using 2D cell cultures in Petri dishes are not equivalent to animal trials. 3D tissue cultures may overcome fundamental obstacles in the development of new therapeutic agents. Many new candidates of therapeutic agents are intended as agonists or antagonists of specific receptors on human cells. For these substances, organ-like test systems based on human cells are mandatory. In some cases, new pharmaceuticals lead to unexpected adverse reactions even after successful animal trials. It is assumed that 3D test systems based on human cells might help to overcome these problems.

## Materials and methods

**Figure 1:**
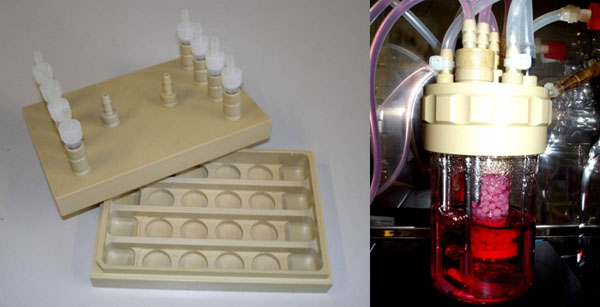
Cultivation systems for 3D-culture of HepG2-cells (A) Multi-well flow-chamber bioreactor (medorex) (B) 10 mL fixed bed reactor (medorex) [1]

Human hepatoma HepG2 cell line was cultivated in monolayer culture and on two 3D carrier systems macroporous ceramic carrier Sponceram (Zellwerk, Germany) and Fibracel composed of polyester non-woven fiber on a polypropylene scaffold (New Brunswick Scientific, USA). 3D-carrier cultivation was performed in 24-well-plates, in a multi-well flow-chamber bioreactor or a fixed bed (Fig. 1) [1]. Cells were seeded at cell densities of 1*10^5^/ml. Medium was DMEM/Ham´s F-12 mixture supplemented with 10% FBS. Growth of cultures was determined using DNA measurement with H33528 after digesting the cells with Papain.An average DNA content of 14.8 pg DNA per cell was previously obtained using defined cell concentrations. Functional assays were carried out according to the method described in [2] using 7-ethoxyresorufin as a substrate. Induction of EROD activitiy was done using 3-methyl-cholanthrene or Ketoconazole. Cellular viability was monitored using Resazurin and live/dead staining (AO/PI).

## Cell growth on carrier systems

On Sponceram, cells spreaded initially but formed dense clusters after extended cultivation. On FibraCel, cells formed small aggregates after seeding. Later they grew within the whole carrier structure. In the flow chamber 3.83 · 10^5^ ± 6.04 · 10^4^ cells per carrier were reached within two compared to 1.45 · 10^6^ ± 3.68 · 10^6^ in 24 well plates. For the fixed bed an average of approx. 1.35 · 10^6^ cells per carrier were obtained.

## Liver specific EROD assay

The cultivation of Hep G2 cells in the two reactor systems was carried out for 7 days and for 14 days prior to induction of EROD activity.. Measurement of EROD activity found to be linear for at least for 1h (sampling every 15 min). Activities were similar to static cultures in the flow chamber [approx. 1.7 fmol Resorufin/(cell*h)]. Lower activities were detected in the fixed bed bioreactor after 14d [approx. 0.5 fmol Resorufin/(cell*h)] compared to 7d [approx. 0.15 fmol Resorufin/(cell*h)], possibly as a result of the formation of large cell clusters.

## Conclusions

In this study it was shown that 3D dynamic culture systems can be used to carry out functional assays such as the EROD assay with a human liver cell line. HepG2 cells could be cultivated for a longer time in dynamic 3D culture and showed a better viability compared to static monolayer cultivation.
